# Analysis of a super-transmission of SARS-CoV-2 omicron variant BA.5.2 in the outdoor night market

**DOI:** 10.3389/fpubh.2023.1153303

**Published:** 2023-07-04

**Authors:** Mingyu Luo, Shelan Liu, Liebo Zhu, Fengying Wang, Kunyang Wu, Hanqing He, Xiaohua Qi, Zhifeng Pang, Xuanjun Dong, Zhenyu Gong, Min Yu

**Affiliations:** ^1^Department of Communicable Disease Control and Prevention, Zhejiang Provincial Center for Disease Control and Prevention, Hangzhou, China; ^2^Yiwu County-level Center for Disease Control and Prevention, Jinhua, China; ^3^Jinhua Prefecture-level Center for Disease Control and Prevention, Jinhua, China; ^4^Department of TB Control and Prevention, Zhejiang Provincial Center for Disease Control and Prevention, Hangzhou, China; ^5^Department of Immunization Program, Zhejiang Provincial Center for Disease Control and Prevention, Hangzhou, China; ^6^Department of Public Health Emergency Response, Zhejiang Provincial Center for Disease Control and Prevention, Hangzhou, China; ^7^Zhejiang Provincial Center for Disease Control and Prevention, Hangzhou, China

**Keywords:** COVID-19, aerosol transmission, omicron, outbreak, aerosol suspension time

## Abstract

**Introduction:**

The COVID-19 pandemic continues to ravage the world, and mutations of the SARS-CoV-2 continues. The new strain has become more transmissible. The role of aerosol transmission in the pandemic deserves great attention.

**Methods:**

In this observational study, we collected data from market customers and stallholders who had been exposed to the virus in the Qingkou night market on July 31 and were subsequently infected. We analyzed the possible infection zones of secondary cases and aerosol suspension time in ambient air. We described and analyzed the characteristics of the secondary cases and the transmission routes for customers.

**Results:**

The point source outbreak of COVID-19 in Qingkou night market contained a cluster of 131 secondary cases. In a less-enclosed place like the Qingkou night market, aerosols with BA.5.2 strain released by patients could suspend in ambient air up to 1 h 39 min and still be contagious.

**Conclusion:**

Aerosols with viruses can spread over a relatively long distance and stay in ambient air for a long time in a less enclosed space, but shorter than that under experimental conditions. Therefore, the aerosol suspension time must be considered when identifying and tracing close contact in outbreak investigations.

## 1. Introduction

In addition to droplet and contact transmission, aerosol transmission has been confirmed as a critical transmission route for COVID-19. Previous studies have detected airborne severe acute respiratory syndrome coronavirus-2 (SARS-CoV-2) in outdoor environment (1, 2). Field investigations have also revealed aerosol transmission linked to COVID-19 clusters in enclosed places, such as department stores or floor drains (3–6). These studies have shown that SARS-CoV-2 can be released into the surrounding air through the patient’s respiratory tract activities (such as coughing, breathing, and speaking) to form droplets of different particle sizes, and can exist for a long time in a relatively enclosed place (3, 4, 7). Until now, the effectiveness of aerosol transmission of the Omicron strain has not been investigated.

Aerosol transmission of SARS-CoV-2 is more likely to occur in enclosed spaces. In an enclosed place with enclosed spatial form of walls and roofs and nearly stationary air flow, virus released from source of infections can persist and accumulate, and susceptible people can be exposed to high concentration of virus and get infected. Besides size range and enclosed spatial form of walls and roofs, we also need to consider the microclimate and environment. In the real world, aerosols with viruses released from patients can remain alive in ambient air, and then cause infection among susceptible people who inhale the aerosols, this suspension time which can result in transmission of COVID-19 should be considered as effective aerosol suspension time. The aerosol suspension time in ambient air in a complex real-world environment determines the ability of the virus to spread. Under experimental enclosed conditions, the measured half-life of SARS-CoV-2 in aerosols can reach approximately 3 h (8, 9). Unlike the half-time of SARS-CoV-2 in experimental conditions, the effective aerosol suspension time in the real world is difficult to measure.

A cluster of more than 100 infections was identified to be related to the Qingkou night market in the Zhejiang Province. The environment of night market is complicated, presenting intersections of relatively open environments, dense customer flow, microclimates, and other factors. In such a severe COVID-19 outbreak, it is essential to analyze the transmission routes.

We retrospectively analyzed the possible transmission routes of the database on infections of the Qingkou night market cluster outbreak to explore aerosol transmission routes in a non-tightly enclosed space and to estimate the suspension time of real-world aerosols in ambient air.

## 2. Materials and methods

### 2.1. Study design and setting

During the prevention and control of this outbreak, the Centers for Disease Control and Prevention (CDCs) and Public Security Bureau (PSBs) oversaw epidemic analysis and strategy assessment. Staff in the CDCs and PSBs were responsible for the epidemiological investigations of infections. To identify infections’ sources of infection and close contacts, we conducted epidemiological interviews with infections and their guardians. We conducted a field investigation on the specific areas in the night market where infections had visited.

Environmental samples in stalls were collected on August 2, and samples in the public washroom were collected on August 7. According to the Technical Guidelines for the Collection and Testing of 2019 Coronavirus Specimens in China, the sampling swab was fully infiltrated with the virus preservation solution and then was applied repeatedly on the surface of sampling sites, the swab was put back into the sampling tube for infiltration and was applied again. This collection process was repeated more than three times for one sampling site. The sampling sites included surface of the stalls where index cases (ICs) had shopped or stayed, surface of facilities in public washroom (doorknob, sink, urinal pools, and toilet seat). These samples were then subjected to SARS-CoV-2 Quantitative Real-time polymerase chain reaction (PCR) testing (Agilent 7,500) using the 2019-n CoV-2 nucleic acid detection kit (Wuhan Easy Diagnosis Biomedicine Co., Ltd.).

### 2.2. Variables

We collected demographic characteristics of infections, information on nucleic acid tests, clinical information, schedule in the last 4 days before diagnosis of COVID-19, close contacts in the last 4 days before diagnosis of COVID-19, and transaction records in the Qingkou night market on July 31. To protect infections’ privacy, we omitted the infections’ personal identities and replaced them with serial numbers.

### 2.3. Participants

We defined the study population as COVID-19 infections who were in direct contact with the Qingkou night market on July 31. We went through every infection’s records to screen for patients who had been exposed to the Qingkou night market. The ICs were identified as N1, N2, and N3; these were confirmed COVID-19 patients who introduced an epidemic in the Qingkou night market.

Secondary cases were customers and stallholders who had been exposed to the Qingkou night market on July 31 and were infected. Exclusion criteria included: (1) individuals who had been to districts where COVID-19 cases were reported within 14 days before being exposed to the Qingkou night market on July 31; (2) individuals who had close contact with COVID-19 infections or infections’ close contacts within 14 days before being exposed to the Qingkou night market on July 31; and (3) individuals who had long-term symptoms such as chronic pharyngitis, fever, sore throat, and other symptoms of respiratory disease within 14 days before being exposed to the Qingkou night market on July 31.

### 2.4. Statistical methods and epidemiological analysis

We described and analyzed the distribution of the characteristics of secondary cases, such as age, sex, and incubation period. We drew the curve for the time of onset of symptoms of cases and analyzed information from the nucleic acid test and the schedule of ICs and secondary cases. Based on the above, we analyzed the sources of infection in secondary cases related to the Qingkou night market.

According to transaction records, we compared the exposure duration of secondary cases in the Qingkou night market and detention time of the ICs to calculate the time intersection. We analyzed the time interval between the secondary cases and ICs’ transaction records to estimate the aerosol suspension time in ambient air. For the same secondary case, he/she could have several transaction records at different times in one specific area where the ICs have been stayed; therefore, different time intervals could be calculated. It is rational that shorter time intervals are more likely to represent higher concentrations of aerosol residues; therefore, we chose the shorter time interval among the different time intervals of one secondary case to calculate the aerosol suspension time.

## 3. Results

### 3.1. Basic information for Qingkou night market

The Qingkou night market is well known among the local population. Function areas contained dining and sundries areas. The dining area (area C) was located in the southwest of the night market, with an area of 190 m × 200 m. The dining area had 69 stalls that sold different types of street food. The Sundries area (areas A and B) was a street with an area of 272 m × 13 m. The Sundries area had 189 stalls with dimensions of 3 m × 1.5 m. Stalls in Area A mainly sold clothes, shoes, hats, and other sundries, whereas stalls in Area B mainly sold clothes (Supplementary Figures S1, S2).

South of the night market, was one building with two floors. The Police Box and Administration Office were on the first floor and a large public washroom was on the second floor.

In the summer, the Qingkou night market opened from 17:00 to 24:00 and could accept approximately 5,000 customers per night. During the day, the stalls were closed, and the aisles in the night market were used for normal traffic.

On the night of July 31, it was cloudy, the temperature was 27°C and the relative humidity was 85%. There was no sustained wind direction, and the wind speed was light air to light breezes. Most of the stalls had big umbrellas.

### 3.2. Index cases

Patient N1 was 40 to 50-year-old, working and living with three family members in Jiangxi and Guangxi Province. N1 went to Zhejiang Province on July 29 with her two family members. On July 31, another immediate family member of N1 was diagnosed as a confirmed COVID-19 patient in Guangxi Province. N1 underwent nucleic acid tests on July 31 and August 1 respectively, and the results were negative. On August 2, N1 underwent a nucleic acid test and was diagnosed with COVID-19. The cycle threshold values of N1 were as follows: N:17.34/ORF:16.71. Phylogenetic analysis revealed that N1 was infected with the BA.5.2 strain. N1 had fever (39°C) on July 30.

N2 and N3 were N1’s immediate family members; N2 had fever (38.5°C) on the afternoon of August 1, and was diagnosed as a confirmed patient on the afternoon of August 3 (N:18.89/ ORF:21.09). N3 had fever in the early morning of August 2, and was diagnosed as a confirmed patient on August 3 (N:16.08/ORF:20.07).

ICs entered the Qingkou night market at 19:17 on July 31 and they stayed in the area of sundries, went to the dining area at 20:05, and left the night market at 20:21.

### 3.3. Demographic characteristics of secondary cases

A total of 131 secondary cases were identified, including 113 customers and 18 stallholders. Among the 131 secondary cases, 79 (60%) were women. The youngest case was 1 year old, and the oldest was 73 years old; the median age of secondary cases was 30 (20, 38) years. There were 64 cases who belonged to 26 families (Table 1).

**Table 1:** Characteristics of the 131 secondary cases in Qingkou night market.

Characteristics	*N*/ Median (Q_1_, Q_3_)	%
Category of cases		
Customer	113	86
Stallholder	18	14
Sex		
Female	79	60
Male	52	40
Age, years	30 (20, 38)	-
Occupation of customers (*N* = 113)		
Worker	32	28
Service/Sales	29	26
Students or preschool children	27	24
Others	25	22
Clinical symptoms had occurred when detected positive by nucleic acid test		
Yes	74	56
No	57	43
Cycle threshold value		
N	26.83 (20.44, 32.78)	-
ORF	26.99 (20.95, 33.20)	-

### 3.4. Analysis of source of infection

Infection status: Owing to the normalized nucleic acid detection strategy, all citizens must perform nucleic acid tests once a week. For at least 2 weeks before patient N1 was diagnosed with COVID-19, no COVID-19 infection was identified in the local district.

Infectious ICs: N1-3 had been to a restaurant at 21:49 of July 29, and the owner (also as a server) of the restaurant was diagnosed as a confirmed patient on August 2. The owner had another source of infection. While the ICs were in the Qingkou night market on July 31, N1 reported that she wore a mask when they stayed in the sundries area, but removed the mask in the dining area. N2 and N3 did not wear masks during the entire duration of the Qingkou night market.

**Figure 1:**
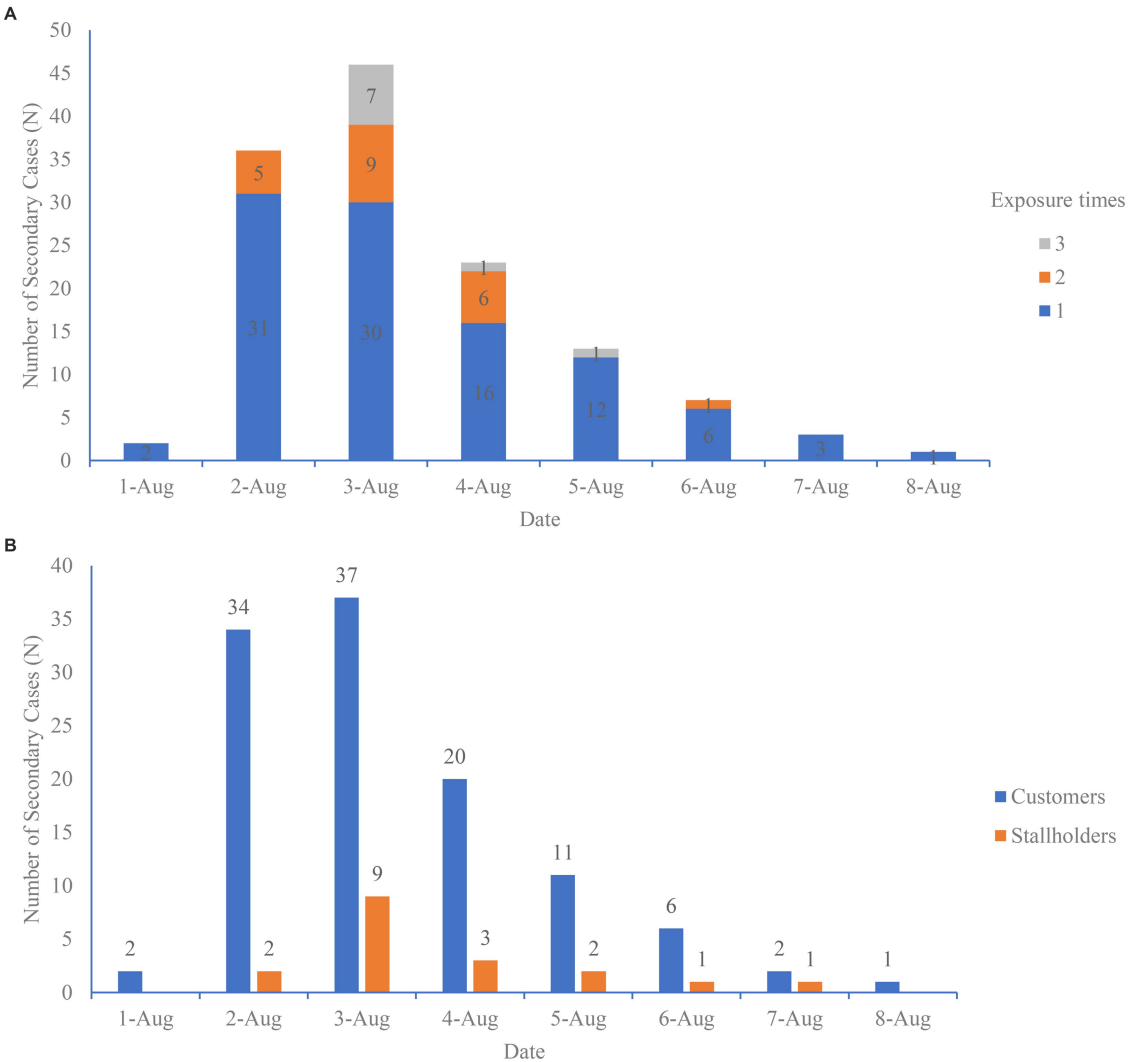
**(A)** Date of onset of symptoms/detection positive by nucleic acid test of 131 secondary cases in the Qingkou night market by exposure times. **(B)** Date of onset of symptoms/detection positive by nucleic acid test of 131 secondary cases in the Qingkou night market by category of cases.

Distribution of the onset of symptom/detection positive by nucleic acid testing of infections: among the 131 infections, all had been exposed to the Qingkou night market on July 31; 21 patients had been exposed to the night market twice (on July 31 and August 1 or on July 31 and August 2); and 9 patients had been exposed to the night market three times (on July 31, August 1, and August 2; Figure 1A). The distribution of the onset of symptom/detection positive by nucleic acid test showed that two secondary cases experienced symptoms on August 1 and August 2, and the number of cases increased rapidly, reaching a peak on the incidence curve on August 3. The incidence curve showed a unimodal distribution of cases, regardless of customer or stallholder (Figure 1B).

Detection of environmental samples: The CDC conducted two detections in different areas, 15 sites of environmental samples were obtained prior to the disinfection process (on August 3) in stalls of the Qingkou night market on the night of August 2; another 40 sites of environmental samples were obtained after the disinfection process (on August 3) in the public washroom of the night market on the afternoon of August 7. The results showed that all the samples were negative.

#### 3.4.1. Analysis of 18 stallholders’ possible infection zones

During the opening hours, stallholders stayed at their stalls most of the time and could be exposed to droplets from ICs when they stayed or passed by stalls or disseminated aerosols in ambient air. Among the 18 stallholders, 14 worked in the sundries area, five had been in the dining area and had meals, and four stallholders worked in the dining area. Stallholders in the sundries area who had been to the dining area might have also had contact ICs or were exposed to ICs’ droplets or aerosols in the dining area.

#### 3.4.2. Analysis of 113 customers’ possible infection zones

##### 3.4.2.1. Possible infection zone one

**Figure 2:**
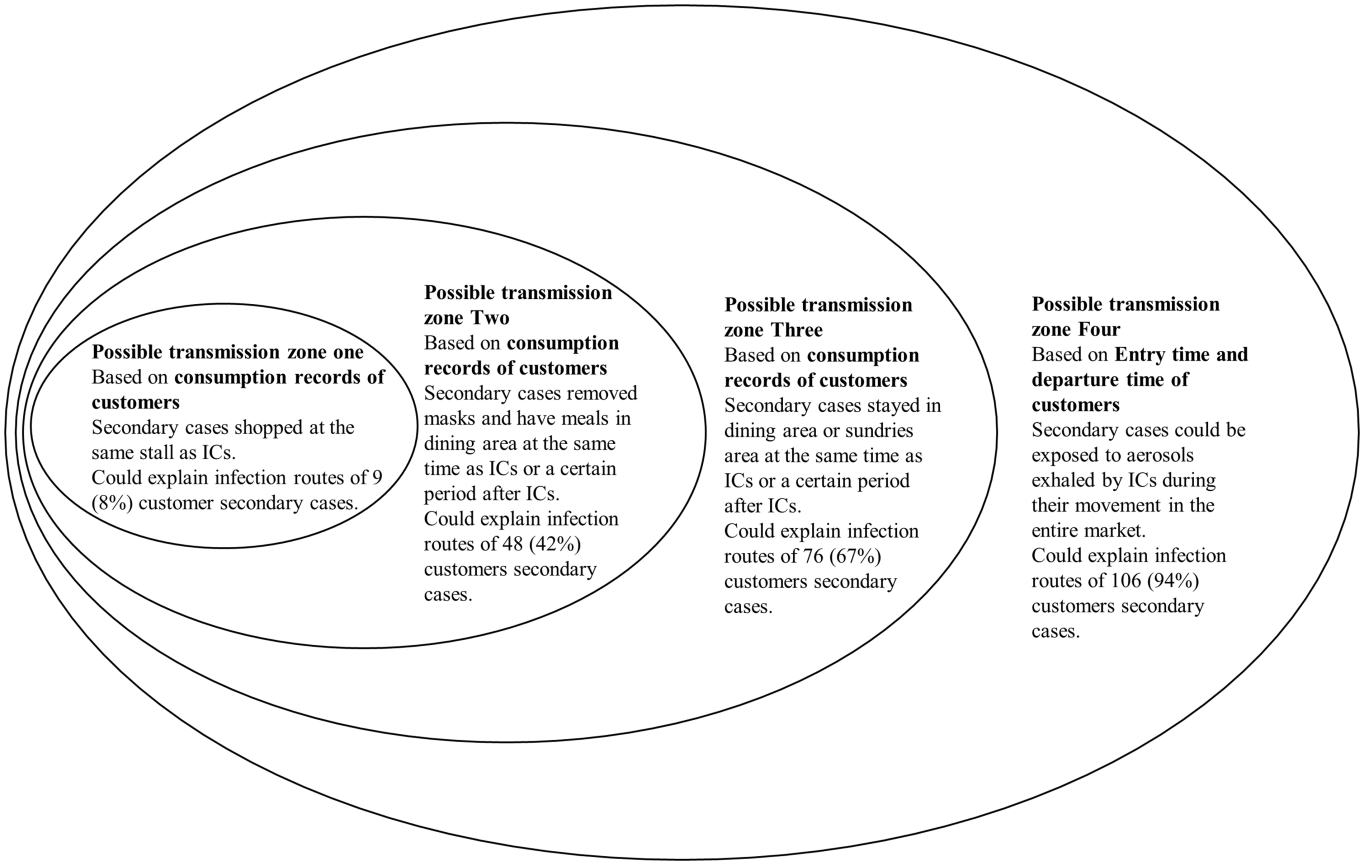
An overview of possible transmission zones one to four.

Within a certain duration, customers shopped at the same stall as ICs, and they could be infected through close proximity with ICs or directly exposed to residual aerosols exhaled by ICs. In the dining area, ICs just purchased food and paid by mobile payment software, then they left specific stalls and ate while walking through the dining area. We considered that the tables, chairs of the specific stalls would be highly unlikely to be contaminated by droplets and aerosols exhaled by ICs. If customers arrived at the same stall after the ICs had left, any chance of close contact at the stall could be eliminated; thus, contact and droplet transmission could be eliminated. Customer infections can be identified as aerosol transmissions (Figure 2).

In total, among the 334 customers, nine customers who shopped at the same stalls as ICs were infected; the incidence of secondary cases was 3% (9/334), and they shopped at the same time or later than ICs (Table 2). N146, N262, and N263 were family members, and had duplicate payment records for stalls B59-60 and A81. For this family, we selected the shorter time interval (41 min) between the payment records of the ICs’ and secondary cases’ as the aerosol suspension time interval. Therefore, the aerosol suspension time was at least 5 min, and up to 41 min.

**Table 2:** Payment records and incidence in divided periods of secondary cases in customers.

Stalls	ICs’ payment records	Secondary cases’ payment records	Time interval between ICs’ and secondary cases’ payment records	Incidence of secondary cases in divided periods^a^							
				Incidence of secondary cases	Before ICs’ payment	0–10 min after ICs’ payment	11–20 min after ICs’ payment	21–30 min after ICs’ payment	31–40 min after ICs’ payment	41–50 min after ICs’ payment	51 min and more after ICs’ payment
B63-64 (Clothes)	19:32	19:46	14 min (N6, N8)	2/23	0/6	0/1	2/3	0/2	0/1	0/2	0/8
B59-60 (Clothes)	19:43	20:31	48 min (N146, N262, N263)	3/24	0/4	0/0	0/1	0/0	0/1	3/4	0/14
A36 (Clothes)	19:56	None	–	–		–	–	–	–	–	–
C57 (Fruity Mix)	20:15	20:15	0 min	2/84	0/30	2/9	0/4	0/5	0/5	0/1	0/60
A81 (Clothes)	19:22	19:27; 20:03	5 min (N7, N249); 41 min (N146, N262, N263)	5/81	0/10	2/7	0/4	0/5	3/5	0/5	0/45
B83 (Accessories)	19:23	None	–	0/65	0/8	0/1	0/4	0/1	0/2	0/0	0/49
B79 (Shoes)	19:24	None	–	0/2	0/0	0/0	0/0	0/0	0/0	0/0	0/2
B72	19:25	None	–	0/12	0/0	0/0	0/0	0/1	0/0	0/0	0/11
B71	19:26	None	–	0/8	0/1	0/0	0/0	0/0	0/0	0/0	0/7
B62	19:33	None	–	0/39	0/9	0/0	0/1	0/1	0/1	0/0	0/27
Total	–	–	–	9/334^b^	0/68	4/18	2/17	0/15	3/15	3/12	0/223

a Stallholders in the sundries area were eliminated.

b N146, N262, and N263 were family members and shopped in B59-60 and A81, respectively. Duplicate data were deleted while counting the total number of secondary cases and customers.

##### 3.4.2.2. Possible infection zone two

Compared to the other functional zones, the dining area was much smaller. When customers removed masks and consumed meals at the same time as ICs or a certain period after ICs, they could be exposed to aerosols exhaled by ICs and become infected. If customers arrived at the dining area after the ICs had left, contact and droplet transmission could be eliminated and aerosol transmission could be identified (Figure 2).

We obtained the transaction records for 64 secondary cases in the dining area. Among them, the payment and dining times of 35 secondary cases intersected with the duration of time that ICs spent in the dining area.

**Figure 3:**
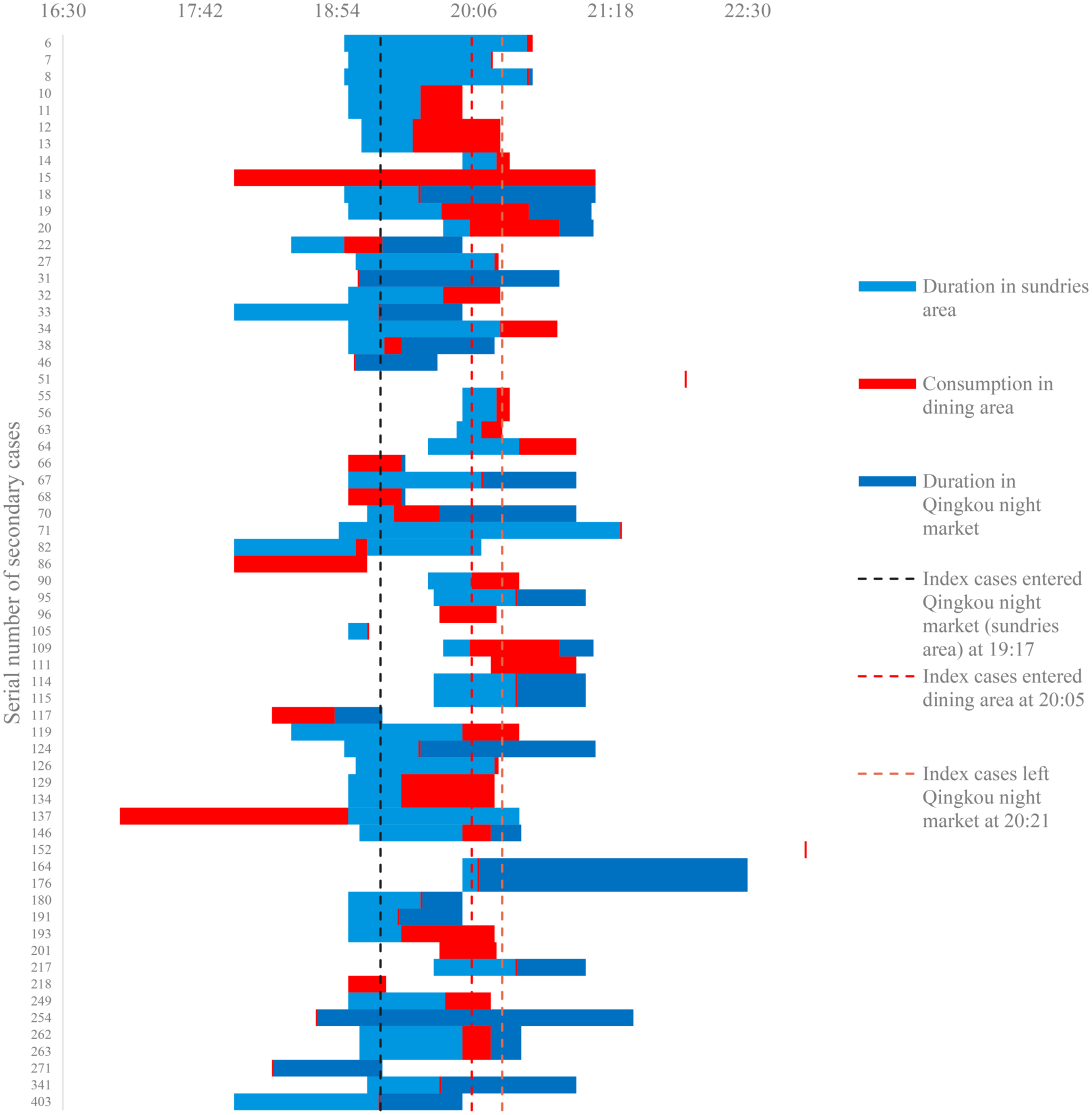
Duration of 64 secondary case with transaction records in dining area.

There were 13 secondary cases whose dining times were later than the ICs’ departure times. The longest time interval (2 h 39 min) was for N152, who was the husband of N130. The couple visited different areas of the night market at different times on July 31. N130 had fever on August 2. N152 was detected as positive by nucleic acid testing in August 5, without any clinical symptoms. The possibility of transmission between family members cannot be ignored. If we consider that N152 was infected by N130 via living together and exclude this outlier, the time interval was at least 4 min, and up to 1 h 39 min (Figure 3).

Possible infection zone two could not explain why 16 secondary cases had already left the Qingkou night market before the ICs entered the dining area.

##### 3.4.2.3. Possible infection zone three

Customers could not only be exposed to aerosols exhaled by ICs at stalls in the dining area but also be exposed at stalls in the area of sundries. If customers arrived in a specific area after the ICs had left, contact and droplet transmission could be eliminated, and aerosol transmission could be identified (Figure 2).

During the collection of infections’ transaction records, we obtained 76 secondary cases with definite transaction records from the Qingkou night market. Among them, 12 secondary cases only had transaction records in sundries area, and all their payment time had intersections with ICs’ duration in sundries area; 36 secondary cases only had transaction records in dining area, and eight of them had meals later than ICs’ departure time; 28 secondary cases had transaction records in sundries area and dining area, among them, four cases’ duration in sundries area or dining area had no intersection with that of ICs.

The time interval between these 12 secondary cases and ICs was at least 4 min, and up to 1 h 39 min (Table 3).

**Table 3:** Secondary cases whose duration in a specific area had no intersection with that of the ICs.

Case	Payment records in sundries area	Time interval between ICs’ and secondary cases’ payment records in sundries area	Payment records in dining area	Time interval between ICs’ and secondary cases’ payment records in dining area
N14	–	–	20:25	4 min
N56	–	–	20:25	4 min
N55	–	–	20:25	4 min
N95	–	–	20:28	7 min
N114	–	–	20:28	7 min
N115	–	–	20:28	7 min
N152	–	-	23:00	2 h 39 min
N217	–	–	20:28	7 min
N20	20:20	15 min	20:43	22 min
N51	21:57	1 h 52 min	22:00	1 h 39 min
N109	20:20	15 min	20:43	22 min
N254	20:30	25 min	18:43	–

##### 3.4.2.4. Possible infection zone four

In fact, customers could not only be exposed to aerosols exhaled by ICs at stalls, but also during their movement in the entire market. If customers arrived at the night market after the ICs had left, contact and droplet transmission could be eliminated and aerosol transmission could be identified (Figure 2).

We calculated the duration of secondary cases in the entire market and compared the duration of secondary cases with that of ICs. The results showed that 102 out of 113 customer secondary cases had definite entry and departure times in the night market. Among them, 93 secondary cases had an intersection with the ICs, and nine secondary cases had no intersection with the ICs. The results showed that the time interval could be up to 1 h 39 min (the longest time interval of 2 h 39 min was eliminated) (Supplementary Table S1; Supplementary Figure S4).

Possible infection zone four could not explain why seven secondary cases had already been left before the ICs entered the same area. In addition, eight secondary cases had stayed in the night market during the ICs’ duration, without transaction records; three secondary cases had walked through the night market during the ICs’ duration. According to their description and researchers’ field investigations, it takes 10–20 min to walk through the night market.

## 4. Discussion

In theory, a night market should be considered as an open space because it lacks a clear physical partition. Therefore, with great caution, we analyzed why such a large outbreak occurred in the Qingkou night market. (1) Unlike typical enclosed places, such as elevators and residential stairwells, the environment of the Qingkou night market is more complicated. The aisles became narrow, with densely arranged stalls (most of the stalls had big umbrellas), when the night market opened. The dining area was relatively enclosed, with smaller areas and walls. On the night of July 31, customers could not feel the wind in the night market; thus, the wind speed could be considered as 0.0–0.03 m/s and ambient air was steady and the area poorly ventilated. The Qingkou night market can be identified as a semi-enclosed space without typical physical partitions. (2) Phylogenetic analysis showed that ICs were infected with the Omicron BA.5.2. The effective reproduction number (Re) of BA.4 and BA.5 are 1.19 and 1.21 times of BA.2 respectively, which is mainly due to mutations of spike L452R/M/Q (10). In addition, the Qingkou night market could accept approximately 5,000 customers per night; this dense customer flow also provided a large number of susceptible individuals. (3) The unimodal incidence curve suggested that the outbreak was exposed to the same source of infection. Although 30 individuals were exposed to the night market on August 1 or August 2, no other peaks were observed in the incidence curve. Therefore, it is logical that such a serious outbreak could occur in the Qingkou night market.

The ICs remained in the night market for 1 h and 4 min. They were already infectious before July 31, and could release droplets and aerosols over the entire period via various respiratory activities (such as coughing, sneezing, speaking, and breathing), as N1 had developed clinical symptoms before July 31 (11–13). In such a noisy environment, they inevitably spoke loudly and released significant amounts of droplets and aerosols (14–16). Sima Asadi’s study showed that the particle emission rate during speech was positively related to the loudness of vocalization (16). The moving lines of the ICs and susceptible populations were complicated. Therefore, the main routes of transmission could be via contact, close-range droplets, and aerosols.

In each possible transmission zone, if customers arrived in the same area after the ICs left, any chance of close contact at the stall could be eliminated; thus, contact and droplet transmission could be eliminated. Customer infection could be identified as aerosol transmissions. Possible transmission zone one to three which were based on transaction records, explained 58% of the infection routes. Obviously, many customers had passed by or stayed at specific stalls or aisles without payment for anything. Smaller aerosol particles can travel further in the ambient air. Bourouiba et al’s study showed that aerosols produced by sneezing and coughing can travel as far as 7–8 m (17). Smaller aerosol particles can remain airborne for a longer time before setting by gravity (17, 18).

Nucleic acid testing was not able to identify ICs’ infection status in time. The operation of nucleic acid sampling might have vulnerabilities, and nucleic acid testing might show false negative results due to the mutation of the virus. From possible transmission zones one to four, the details of transaction records gradually decreased, but the number of cases whose infection routes could be explained increased. Each transmission route has its own limitations and unexplained aspects. Although we traced patients’ moving lines using transaction records via payment software and investigation results, recall bias was inevitable. The environmental samples were collected at least 2 days after the outbreak in the night market and were all negative; therefore, it was difficult to verify the range of aerosol dissemination. SARS-CoV-2 could be stable in a short time in the night market environment with a relatively high temperature (19) and adequate ventilation in the daytime. Our conclusion that this outbreak was a point source outbreak also confirmed that no residual aerosol existed in ambient air or on the surface of stalls after July 31.

This outbreak of COVID-19 in Qingkou night market has confirmed that aerosols can spread further and stay in ambient air for a long time in a place that is not strictly enclosed, but shorter than that in experimental conditions. In a less-enclosed place like the Qingkou night market, aerosols containing the BA.5.2 strain released by patients could suspend in ambient air for up to 1 h 39 min and still be contagious. The aerosol suspension time in air must be considered to expand the time range in the process of identifying close contacts.

In addition, the mutation of the SARS-CoV-2 continues, and its transmission ability is increasing (20–23). Moreover, the limitations of regular personal physical containment strategies (such as wearing masks) have gradually appeared. The global pandemic of COVID-19 still persists and it is important to discover more technical details like aerosol suspension time for normalized prevention and control strategies.

## Data availability statement

The data analyzed in this study is subject to the following licenses/restrictions: Original database contains participants’ personal information, which is not publicly available. Requests to access these datasets should be directed to zhygong@cdc.zj.cn.

## Ethics statement

Ethical review and approval was not required for the study on human participants in accordance with the local legislation and institutional requirements. Written informed consent from the participants’ legal guardian/next of kin was not required to participate in this study in accordance with the national legislation and the institutional requirements.

## Author contributions

ML and SL analyzed the results and wrote the manuscript. LZ, FW, ZP, XD, ZG, and MY organized the epidemiological investigations. HH and XQ conducted the field investigation. KW collected and checked the database. ZG and MY revised the manuscript and guaranteed the article. All authors contributed to the article and approved the submitted version.

## Conflict of interest

The authors declare that the research was conducted in the absence of any commercial or financial relationships that could be construed as a potential conflict of interest.

## Publisher’s note

All claims expressed in this article are solely those of the authors and do not necessarily represent those of their affiliated organizations, or those of the publisher, the editors and the reviewers. Any product that may be evaluated in this article, or claim that may be made by its manufacturer, is not guaranteed or endorsed by the publisher.
