# Corrigendum: Analysis of a super-transmission of SARS-CoV-2 omicron variant BA.5.2 in the outdoor night market

**DOI:** 10.3389/fpubh.2023.1272251

**Published:** 2023-09-25

**Authors:** Mingyu Luo, Shelan Liu, Liebo Zhu, Fengying Wang, Kunyang Wu, Hanqing He, Xiaohua Qi, Zhifeng Pang, Xuanjun Dong, Zhenyu Gong, Min Yu

**Affiliations:** ^1^Department of Communicable Disease Control and Prevention, Zhejiang Provincial Center for Disease Control and Prevention, Hangzhou, China; ^2^Yiwu County-level Center for Disease Control and Prevention, Jinhua, China; ^3^Jinhua Prefecture-level Center for Disease Control and Prevention, Jinhua, China; ^4^Department of TB Control and Prevention, Zhejiang Provincial Center for Disease Control and Prevention, Hangzhou, China; ^5^Department of Immunization Program, Zhejiang Provincial Center for Disease Control and Prevention, Hangzhou, China; ^6^Department of Public Health Emergency Response, Zhejiang Provincial Center for Disease Control and Prevention, Hangzhou, China; ^7^Zhejiang Provincial Center for Disease Control and Prevention, Hangzhou, China

**Keywords:** COVID-19, aerosol transmission, omicron, outbreak, aerosol suspension time

**Incorrect Affiliation**

In the published article, there was an error in affiliations 2, 3, 4, 5, and 6. The affiliations were incorrectly written as, “^2^Jinhua Prefecture-level Center for Disease Control and Prevention, Jinhua, China, ^3^Department of TB Control and Prevention, Zhejiang Provincial Center for Disease Control and Prevention, Hangzhou, China, ^4^Department of Immunization Program, Zhejiang Provincial Center for Disease Control and Prevention, Hangzhou, China, ^5^Department of Public Health Emergency Response, Zhejiang Provincial Center for Disease Control and Prevention, Hangzhou, China, ^6^Yiwu County-level Center for Disease Control and Prevention, Jinhua, China”, it should be, “^2^Yiwu County-level Center for Disease Control and Prevention, Jinhua, China, ^3^Jinhua Prefecture-level Center for Disease Control and Prevention, Jinhua, China, ^4^Department of TB Control and Prevention, Zhejiang Provincial Center for Disease Control and Prevention, Hangzhou, China, ^5^Department of Immunization Program, Zhejiang Provincial Center for Disease Control and Prevention, Hangzhou, China, ^6^Department of Public Health Emergency Response, Zhejiang Provincial Center for Disease Control and Prevention, Hangzhou, China”.

**Incorrect Author Name**

In the published article, the authors' names were incorrectly written as Luo Mingyu^1†^, Liu Shelan^1†^, Zhu Liebo^2†^, Wang Fengying^2†^, Wu Kunyang^3^, He Hanqing^4^, Qi Xiaohua^5^, Pang Zhifeng^2^, Dong Xuanjun^6^, Gong Zhenyu^1*^ and Yu Min^7*^. The correct spellings are Mingyu Luo^1†^, Shelan Liu^1†^, Liebo Zhu^2†^, Fengying Wang^3†^, Kunyang Wu^4^, Hanqing He^5^, Xiaohua Qi^6^, Zhifeng Pang^3^, Xuanjun Dong^2^, Zhenyu Gong^1*^ and Min Yu^7*^.

The authors apologize for these errors and state that this does not change the scientific conclusions of the article in any way. The original article has been updated.

