# Supplementary material for: Analysis of a super-transmission of SARS-CoV-2 omicron variant BA.5.2 in the outdoor night market
Source: Front Public Health. 2023 Jul 4;11:1153303. doi: 10.3389/fpubh.2023.1153303 (PMC10352652; doi:10.3389/fpubh.2023.1153303)
Supplement: Supplementary file 1 [file Table_1.DOCX]

**S4 Table 2 Secondary cases whose duration in whole Qingkou night market had no intersection with that of ICs**

| **NO.** | **Entry time** | **Consumption records in Qingkou night market** | **Departure time** | **Date of onset of symptom/detected positive by nucleic acid test** | **Possible intersection with ICs** | **Other possible sources of infection** |
| --- | --- | --- | --- | --- | --- | --- |
| N51 | 21:57 | Dining area:  shopped at C52 stall (steamed cold noodles) at 22:00· | 22:00 | Aug 3^rd^ |  | None |
| N66 | 19:00 | Dining area:  shopped at HAOYISHAO stall (baked oysters) at 19:00;  shopped at YUSHAN wishbone stall at 19:06;  shopped at XIAOLINZI handmade steamed cold noodle stall at 19:07;  shopped at GUOGOUPU stall (nuts and snacks) at 19:28· | 19:30 | Aug 3^rd^ | Departure time has intersection with ICs’ duration in sundries area | N68 and N68 belonged to same family |
| N68 | 19:00 | Dining area:  shopped at HAOYISHAO stall (baked oysters) at 19:00;  shopped at YUSHAN wishbone stall at 19:06;  shopped at XIAOLINZI handmade steamed cold noodle stall at 19:07;  shopped at GUOGOUPU stall (nuts and snacks) at 19:28· | 19:30 | Aug 3^rd^ | Departure time has intersection with ICs’ duration in sundries area | N68 and N68 belonged to same family |
| N73 | 17:00 | Not clear | 18:40 | Aug 3^rd^ | Might contact ICs outside the night market | None |
| N80 | 18:00 | Not clear | 19:00 | Aug 5th | might contact ICs outside the night market | None |
| N86 | 18:00 | Dining area, exact consumption records were not clear | 19:10 | Aug 3^rd^ | Departure time has intersection with ICs’ duration in sundries area | None |
| N152 | 23:00 | Dining area:  shopped at C10 stall at 23:00 |  | Aug 5th |  | N152 and N130 belonged to same family |
| N167 | 18:40 | Not clear | 19:15 | Aug 4th | Might contact ICs outside the night market | N167, N146, N262, and N263 belonged to same family |
| N218 | 19:00 | Dining area:  shopped at GUMING Milk tea stall, exact consumption records were not clear | 19:20 | Aug 4th | Departure time has intersection with ICs’ duration in sundries area | None |
