# Supplementary figures and images for: Analysis of a super-transmission of SARS-CoV-2 omicron variant BA.5.2 in the outdoor night market
Source: Front Public Health. 2023 Jul 4;11:1153303. doi: 10.3389/fpubh.2023.1153303 (PMC10352652; doi:10.3389/fpubh.2023.1153303)

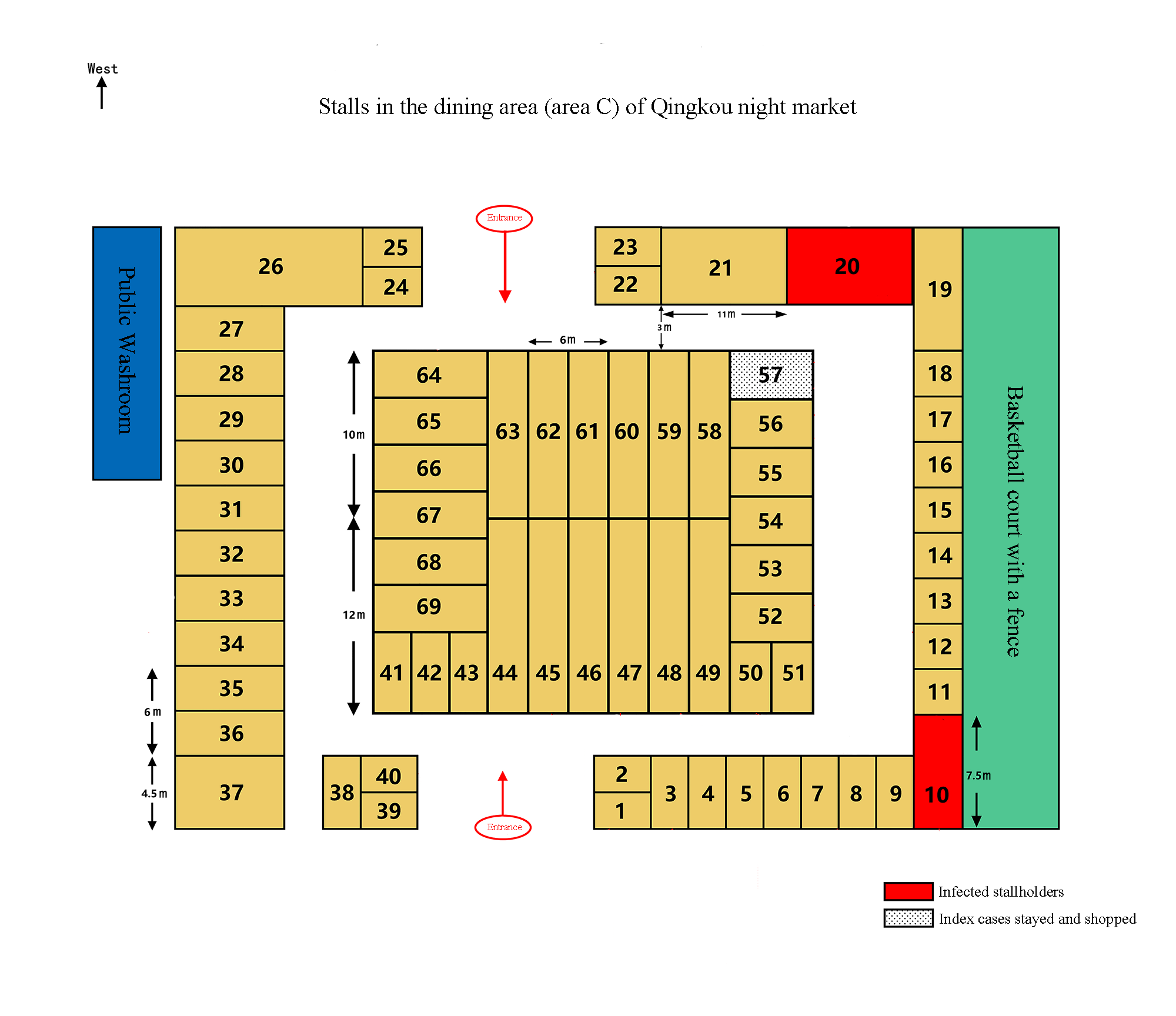

Supplement: Supplementary file 2 [file Image_1.TIF]

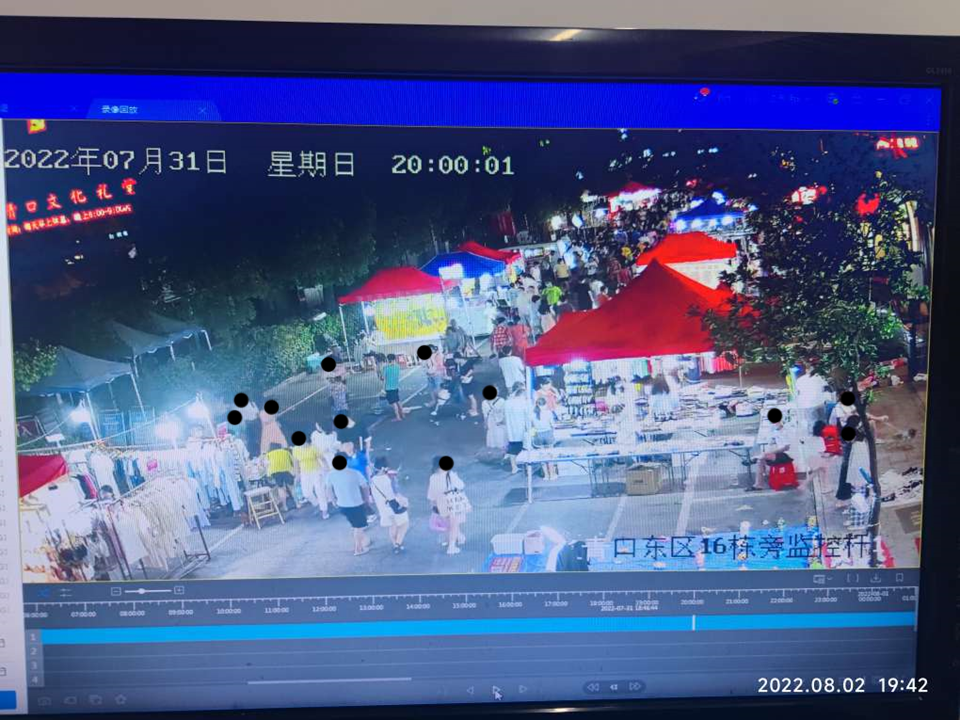

Supplement: Supplementary file 4 [file Image_3.PNG]

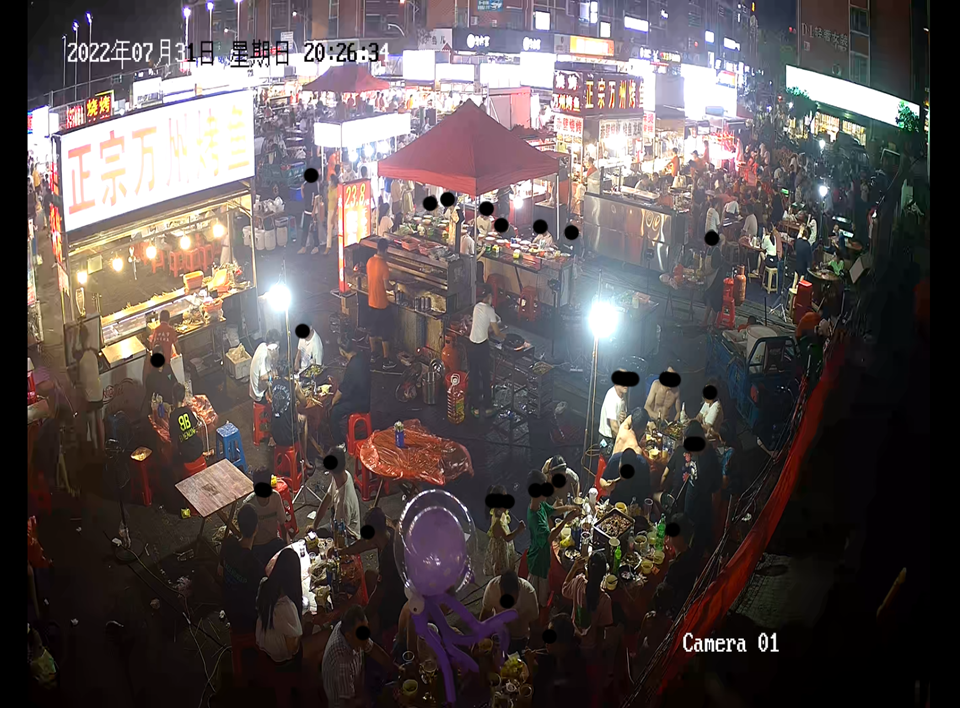

Supplement: Supplementary file 5 [file Image_4.PNG]
